# Spin pumping in magnetic trilayer structures with an MgO barrier

**DOI:** 10.1038/srep35582

**Published:** 2016-10-18

**Authors:** A. A. Baker, A. I. Figueroa, D. Pingstone, V. K. Lazarov, G. van der Laan, T. Hesjedal

**Affiliations:** 1 Department of Physics, Clarendon Laboratory, University of Oxford, Oxford, OX1 3PU, United Kingdom; 2 Magnetic Spectroscopy Group, Diamond Light Source, Didcot, OX11 0DE, United Kingdom; 3 Department of Physics, University of York, Heslington, York, YO10 5DD, United Kingdom

## Abstract

We present a study of the interaction mechanisms in magnetic trilayer structures with an MgO barrier grown by molecular beam epitaxy. The interlayer exchange coupling, *A*_*ex*_, is determined using SQUID magnetometry and ferromagnetic resonance (FMR), displaying an unexpected oscillatory behaviour as the thickness, *t*_MgO_, is increased from 1 to 4 nm. Transmission electron microscopy confirms the continuity and quality of the tunnelling barrier, eliminating the prospect of exchange arising from direct contact between the two ferromagnetic layers. The Gilbert damping is found to be almost independent of the MgO thickness, suggesting the suppression of spin pumping. The element-specific technique of x-ray detected FMR reveals a small dynamic exchange interaction, acting in concert with the static interaction to induce coupled precession across the multilayer stack. These results highlight the potential of spin pumping and spin transfer torque for device applications in magnetic tunnel junctions relying on commonly used MgO barriers.

Much attention is currently devoted to the study of spin-transfer torque (STT)^1^,^2^,^3^,^4^, through which it is possible to realize spontaneous magnetization precession and switching. Spin pumping from a ferromagnet (FM) into a non-magnetic (NM) material is one of the most promising candidates for these applications^5^. Spin pumping has been under intense scrutiny since it was first proposed in 2002^6^, studying the generation of pure spin currents by ferromagnetic resonance (FMR). Of particular importance is the transmission of spins across NM barriers, such as the conductors Ag (ref. 7), Au (ref. 8), and Cu (ref. 9), or insulators like MgO (ref. 10) and SrTiO_3_ (ref. 11). The efficacy of spin pumping is governed by the spin diffusion length of the NM layer, and the spin mixing conductance of the FM/NM interface^12^. Spin pumping is heavily suppressed in insulators, leading to very short spin-coherence lengths, often under a nm^11^. However, coupling of the spin and charge degrees of freedom, and associated charge pumping in tunnelling heterostructures with an insulating barrier, can complicate interpretation of the results^5^,^13^,^14^. It is useful to investigate spin pumping through insulating layers using probes that are insensitive to charge-based effects.

Here, we present a study of spin pumping and static exchange coupling in MgO-based magnetic heterostructures grown by molecular-beam epitaxy (MBE). These magnetic tunnel junctions (MTJs) are well-established high tunnelling magnetoresistance ratio elements^15^,^16^. The strength and character of the interactions is determined in Co_50_Fe_50_ /MgO/Ni magnetic heterostructures using superconducting quantum interference device (SQUID) magnetometry and vector network analyser (VNA) FMR. The structures are of high crystalline quality, as demonstrated by transmission electron microscopy (TEM). Static interlayer exchange is observed through shifts in the resonant field. X-ray detected FMR (XFMR) measurements confirm the presence of static exchange coupling, but demonstrate that for the thinnest MgO barrier there is also a component of spin pumping. These results show the importance of spin transfer in technologically-relevant MTJs, demonstrating that torques can be achieved for suitably thin barriers.

## Ferromagnetic Resonance

In the classical limit the spin dynamics of a ferromagnet are governed by the damped Landau-Lifshitz-Gilbert (LLG) equation of motion, describing the precession of the magnetisation about an effective field arising from internal and external fields:


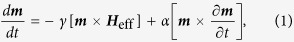


with *γ* the gyromagnetic ratio, ***m*** is the unit magnetisation vector of the material, *α* the dimensionless Gilbert damping parameter, and ***H***_eff_ the effective magnetic field. Through ***H***_eff_ the various energy terms, such as the exchange energy, the demagnetisation energy, the in-plane cubic and uniaxial anisotropies, the static exchange, and the Zeeman energy enter the equation.

The static interlayer exchange coupling is a general term for any interaction that acts to (anti-)align the magnetisations of the two layers in a magnetic trilayer structure such as a spin valve or an MTJ. Examples of such interactions include Ruderman-Kittel-Kasuya-Yosida (RKKY), superexchange, Néel or orange peel coupling, and direct exchange through a discontinuous spacer layer. The presence of a static interaction modifies the LLG equation with an additional term^17^:


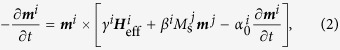


where *β* is the interlayer exchange, and *i*, *j* index magnetic layers. The interlayer exchange is defined as^18^,^19^:


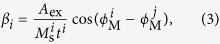


with *A*_ex_ the interlayer exchange constant, *t* the thickness of the magnetic layer, *ϕ*_M_ the equilibrium orientation of the magnetization, and *i*, *j* indices label the magnetic layers. The sign of *A*_ex_ indicates whether the interaction favours parallel (positive) or antiparallel (negative) alignment.

In FMR experiments, additional interaction mechanisms must be considered. As the magnetisation of a ferromagnetic layer precesses on resonance it acts as a spin battery, generating a pure spin current transverse to the axis about which it precesses. When the FM layer is thicker than the ferromagnetic coherence length, a pure spin current can be driven into an adjacent NM layer. A spin current can persist across the spacer layer, and in a trilayer structure either return to the first FM/NM interface, or else flow through to a second NM/FM interface. If it is not reflected here, it crosses the interface and is absorbed by the FM, inducing precession through the STT^4^.

The increased flow of spin momentum out of the FM layer acts as an additional channel for energy loss, leading to an increase in damping. This damping is linear with resonant frequency, and can thus be described in the same terms as Gilbert damping. The absorbed spin current leads to a comparable anti-damping term in the off-resonance layer.

With the addition of these two coupling mechanisms, the LLG for a trilayer system becomes:


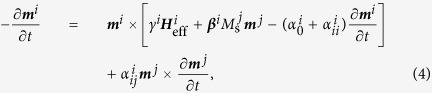


where superscripts denote magnetic layers, and 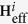 is the effective field acting on layer *i*. The intrinsic Gilbert damping is 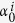, while 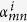 are spin source (*m* = *n*) and spin sink (*m* ≠ *n*) terms. The XFMR results can be modelled using this a linearised solution of this equation.

The presence of these coupling mechanisms alters the magnetodynamics of the on- and off-resonance layer. The static coupling shifts the resonant field of both layers, as it functions as an additional field term in the Kittel equation. Spin pumping, on the other hand, broadens the resonances by providing an additional energy loss mechanism. However, the most important change is the precession induced in the off-resonance layer, wherein the exchange interactions transfer energy between the two layers. This leads to changes in both the phase and amplitude of precession, with the phase being the more sensitive probe.

## Results

### Structural properties of the magnetic trilayer structures

**Figure 1:**
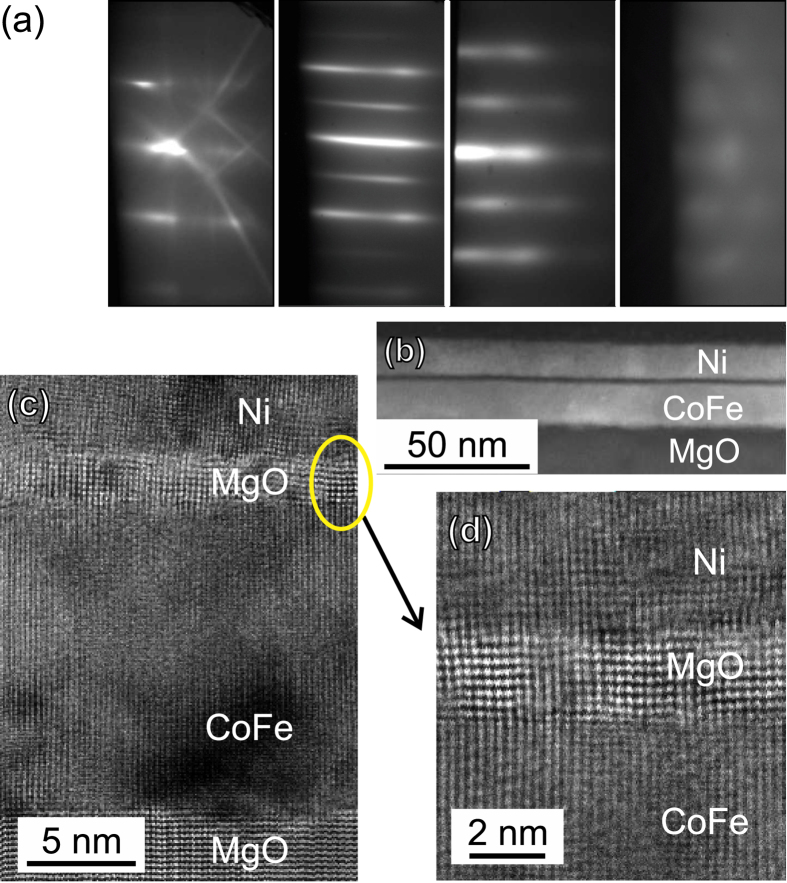
Heterostructure growth and structural properties. (**a**) RHEED images of the MgO substrate (at 700 °C), CoFe layer, 3-nm-thick MgO barrier (after annealing at 300 °C), and Ag-capped Ni layer (from left to right). (**b–d**) Cross-sectional TEM view of the magnetic heterostructure with a 2-nm-thick MgO barrier. (**b**) Low-magnification high angle annular dark field image of the magnetic heterostructure showing uniform thickness of the ferromagnetic layers and the MgO tunnel barrier. (**c**) High-resolution bright-field scanning TEM showing the atomic structure of the substrate, ferromagnetic layers, and barrier viewed along [010]. (**d**) Interface region of ferromagnetic layer(s)/MgO barrier showing the atomically abrupt interfaces and the well-structured MgO barrier textured along the [010] direction.

The structural properties of the grown magnetic heterostructures were studied using transmission electron microscopy (TEM). Figure 1b–d shows cross-sectional TEM images of the magnetic heterostructures. These images demonstrate epitaxial growth of the films, and confirm that the MgO barrier is continuous (down to the thinnest barrier thickness of 1 nm, see Supplementary Fig. S1 for details).

**Figure 2:**
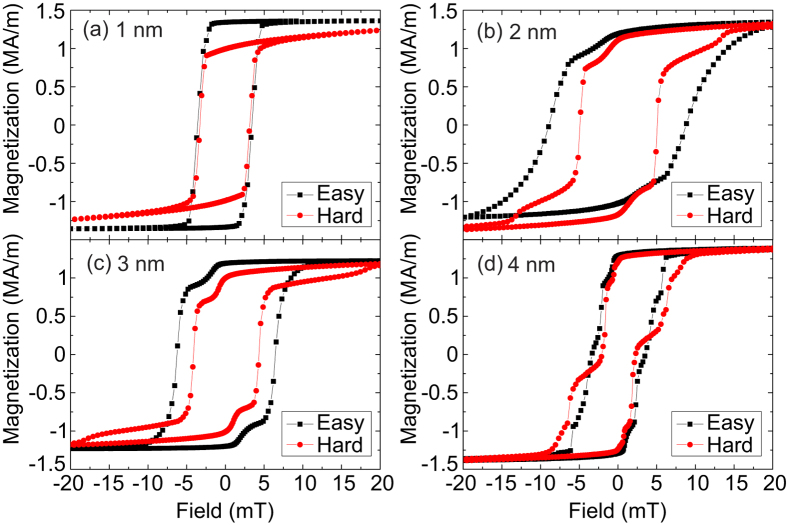
Magnetometry. Hysteresis loops of the magnetic heterostructure with MgO barrier thickness of (**a**) 1 nm, (**b**) 2 nm, (**c**) 3 nm, and (**d**) 4 nm. Strong coupling for the thinnest barrier aligns the magnetizations of the two layers at all fields, but as barrier thickness increases they start to move independently, leading to two distinct steps in (**d**).

Figure 2 shows hysteresis loops measured by SQUID-VSM for all four magnetic heterostructures, displaying a reduction in static interlayer coupling as a function of increasing *t*_MgO_. For the thinnest MgO barrier (*t*_MgO_ = 1 nm, Fig. 2a) the two layers are strongly bound, and there is a single switching step, with a coercive field of 2 mT. As the thickness of the barrier increases, the layers decouple and behave independently. For *t*_MgO_ = 2 and 3 nm (Fig. 2bc), the coupling between the two layers appears to cause winding, leading to a smeared out transition as opposed to sharp steps. Nevertheless, two distinct steps in the hysteresis loop can be identified.

As the TEM measurements show that the MgO barrier appears to be continuous, the observed coupling cannot be due to direct, large-area contact between the two ferromagnetic layers, but it is more likely to be resulting from tunnelling across the barrier. However, there is some evidence of surface roughness of the MgO, which could lead to a Néel orange-peel coupling.

### Lab-based FMR measurements

VNA-FMR measurements were performed to determine values for the interlayer exchange coupling and magnetocrystalline anisotropy parameters for all samples. Figure 1a shows a representative field-frequency map for the sample with *t*_MgO_ = 1 nm. Fits to resonance fields were performed as a function of RF frequency and magnetization alignment using the Kittel equation^20^, including the interlayer exchange field^17^,^18^, using Eq. (3).

**Figure 3:**
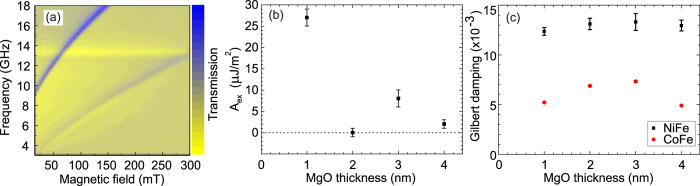
Lab-based FMR measurements. VNA-FMR results for the strength of static exchange coupling and spin pumping: (**a**) Field-frequency transmission map for the sample with *t*_MgO_ = 1 nm, and the magnetic field applied along the easy axis of the CoFe. (**b**) Interlayer exchange coupling, *A*_ex_, as a function of MgO barrier thickness extracted from frequency-dependence of the resonance field, using the Kittel equation. (**c**) Gilbert damping extracted from linewidth of resonance using Eq. (5).

The extracted interlayer coupling is shown in Fig. 3b, while values for all the fitting parameters are given in Table 1. The complete suppression of the interaction at *t*_MgO_ = 2 nm is surprising, as it suggests an oscillatory coupling strength, a phenomenon more often associated with coupling across NM conductive layers (see, e.g., ref. 21). This finding is in contrast to previous work on MgO, e.g., ref. 18. Note that in spin-dependent tunnelling such an oscillatory thickness-dependence has been observed for MgO barriers in Fe/MgO/Fe tunnelling structures^22^.

The total Gilbert damping was extracted from the frequency dependence of the linewidth, Δ*H*, using^23^


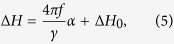


with Δ*H*_0_ the extrinsic broadening. Results for both FM layers are plotted as a function of MgO barrier thickness in Fig. 3c. Within the error bars, there is no change in the Gilbert damping of the Ni layer, which supports the assertion that an insulating layer such as MgO does not permit a spin current to flow. The data for the CoFe layer is more complicated, showing at first a slight increase, then a drop in damping. Microscopy and magnetic characterization gave no evidence of a change in the structural or magnetic properties of the CoFe layer to account for this finding.

While a drop in damping with increasing interlayer thickness is usually indicative of spin pumping, the trend here does not match the exponential decay observed in previous studies^7^,^11^,^24^. Measurements on comparable bare CoFe layers yielded a Gilbert damping of ~3 × 10^−3^. This suggests that at least some of the spins pumped by the precessing magnetization of the CoFe are absorbed. The lower damping in the case of the thinnest MgO layer could be in part due to the strong static exchange coupling observed.

### Synchrotron-based FMR measurements

**Figure 4:**
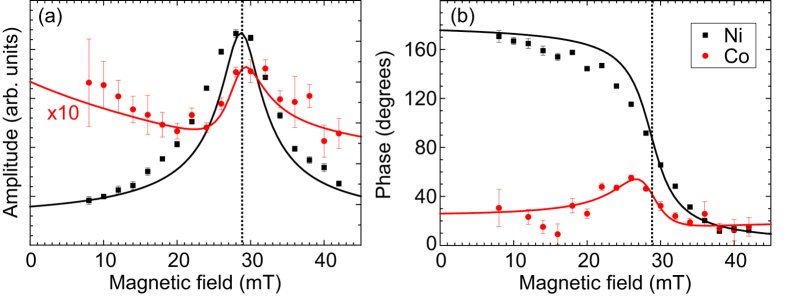
Synchrotron-based FMR measurements. XFMR results for the sample with *t*_MgO_ = 1 nm. (**a**) Amplitude and (**b**) phase of precession of the magnetization at 4 GHz for Ni (black) and Co (red). The magnetic field is applied along the easy axis of the CoFe. The amplitude of induced precession in Co is approximately ten times smaller than that of the Ni; the data has been scaled accordingly. Solid lines are results from a macrospin solution of the coupled LLG equation.

XFMR measurements were performed to study off-resonance precession induced by static exchange coupling and the STT exerted by pumped spins. Figure 4 shows the amplitude and phase of precession at 4 GHz for each layer in the sample with *t*_MgO_ = 1 nm, with the magnetic field applied along the easy axis of the CoFe. The resonance in the Ni layer is easily identified, with a peak in amplitude at 28 mT. Off-resonance precession is induced in the CoFe layer; the resulting dynamic XMCD signal is approximately ten times smaller than that of the Ni. There is also a small phase feature in the Co, which is predominantly unipolar in character. Taken together with the bipolar shape of the Co amplitude variation, this indicates that the static interaction is primarily responsible for induced precession in the CoFe.

Solid lines in Fig. 4 are fits to the data using a macrospin solution of the coupled LLG equation, as outlined in, e.g., refs 9 and 24. The values of static exchange coupling and magnetocrystalline anisotropy determined in Table 1 were used to calculate the amplitude and phase of precession for each layer. In order to reproduce the slightly distorted features in the Co, a dynamic interaction had to be introduced, accounting for 50% of the total Gilbert damping in the Ni layer, and assuming perfect transmission of spins. This modifies the shape of the Co features, mixing the characteristics of the two interactions. With this included the model reproduces the experimental data, although it underestimates the linewidth of the Ni mode due to the presence of inhomogeneous broadening arising from non-Gilbert damping in the samples. The measurement of a component of dynamic interaction shows that a suitably thin MgO barrier will permit a spin current to flow, while for thicker barriers the pumped spin current is entirely scattered within the MgO.

## Discussion

We have presented a study of the coupled magnetodynamics of magnetic heterostructures, demonstrating that both static and dynamic coupling can exist across a suitably thin MgO barrier. SQUID-VSM and VNA-FMR showed the presence of a static interaction acting to align the two layers, with an apparent oscillatory coupling strength — a surprising result that will stimulate further investigation of such structures. Moreover, it was observed that the pure spin current generated by ferromagnetic resonance is absorbed by the MgO, increasing Gilbert damping in the ferromagnetic layers. Layer-resolved XFMR measurements showed that the resulting dynamic interaction can persist across a 1-nm-thick barrier and induce off-resonance precession through spin transfer torque. These results demonstrate how the coupling between magnetic layers can be manipulated through the choice of barrier material and thickness, and illustrate the differing regimes of static and dynamic coupling that can be thus attained. The two interactions can cooperatively transfer energy between layers during ferromagnetic resonance. The pure spin current can persist for a short distance across an insulating barrier, and exert a spin-transfer torque on a neighbouring layer, an important finding for the diverse and active field of STT-MRAM.

## Methods

### Thin film growth

The magnetic multilayer samples were prepared by MBE on epi-ready MgO(001) substrates. Reflection high-energy electron diffraction (RHEED) measurements were performed throughout the growth to monitor crystal quality (cf. Fig. 1a). The full structure is MgO/Co_50_Fe_50_ (15)/MgO(*t*_MgO_)/Ni(8)/Ag(4) (nominal thicknesses in nm), with *t*_MgO_ = 1, 2, 3, and 4 nm (equivalent to ~4.7, 9.5, 14.2, and 19.0 monolayers). The substrates were annealed at 700 °C to improve surface quality, then cooled to 500 °C for the deposition of stoichiometric Co_50_Fe_50_. The samples were further cooled to room temperature for deposition of MgO onto the epitaxial Co_50_Fe_50_ (hereafter referred to as CoFe, for brevity). The MgO barriers were annealed at 300 °C for 20 minutes leading to a sharpening of the RHEED streaks, as shown in Fig. 1a, indicative of good crystalline quality, before being again cooled to room temperature for the deposition of Ni and finally the Ag capping layer.

### Electron microscopy

Structural properties of the grown magnetic heterostructures were studied using a JEOL 2200FS double aberration-corrected (scanning) transmission electron microscope (S)TEM. Cross-sectional TEM specimen were prepared using conventional methods that include mechanical thinning and polishing followed by Ar ion milling in order to achieve electron transparency^25^.

### Magnetometry

Magnetometry was carried out using a SQUID vibrating sample magnetometer (VSM). FMR measurements were performed using a VNA and octupole electromagnet. Real and imaginary components of the microwave transmission parameter, *S*_12_, were measured as a function of magnetic field (strength and angle), and RF frequency. Plotting the resonant field as a function of frequency and bias field angle yields anisotropy and exchange coupling parameters using the Kittel equation^20^ including static exchange coupling^17^,^24^. See the Supplementary Information for a discussion of the Kittel equation.

### X-ray detected Ferromagnetic resonance

Indirect measurements of spin transfer using VNA-FMR are unable to provide a conclusive determination of the presence of spin pumping within the heterostructures. XFMR measurements address this limitation, studying the coupled magnetodynamics with a layer-resolved probe, which reveals more information about the nature of the coupling mechanisms. Further, XFMR can determine whether a pumped pure spin current is absorbed by the MgO barrier, or whether some component crosses it, to be absorbed by the spin sink layer. The XFMR measurements were performed on beamline I10 at the Diamond Light Source (UK) and beamline 4.0.2 at the Advanced Light Source (USA). The magnetic samples are excited by microwave radiation, phase-locked to the synchrotron master oscillator, resulting in a steady precession about the effective field close to the FMR condition. The oscillating magnetization component along the incident x-ray beam direction is probed using x-ray magnetic circular dichroism (XMCD). Element, and consequently layer specificity, is obtained by tuning the x-ray energy to the Ni and Co *L*_3_ edges. For full details of the XFMR methodology, we refer to ref. 26.

## Additional Information

**How to cite this article**: Baker, A. A. *et al*. Spin pumping in magnetic trilayer structures with an MgO barrier. *Sci. Rep.* **6**, 35582; doi: 10.1038/srep35582 (2016).

## Supplementary Material

Supplementary Information

## Figures and Tables

**Table 1:** Magnetocrystalline anisotropy parameters and exchange coupling for the CoFe and Ni layers of the magnetic heterostructures, determined by fitting the angle- and frequency-dependent FMR field for structures with the indicated MgO thicknesses.

FM layer	*t*_MgO_ (nm)	*K*_*c*||_ (kJ/m^3^)	*K*_*u*||_ (kJ/m^3^)	*A*_ex_ (*μ*J/m^2^)
Co_50_Fe_50_	1	48.4 ± 0.3	4.0 ± 0.3	27 ± 2
	2	41.6 ± 0.2	3.2 ± 0.2	0 ± 1
	3	47.5 ± 0.3	1.9 ± 0.3	8 ± 2
	4	43.4 ± 0.2	0.8 ± 0.3	2 ± 1
Ni	1	0.3 ± 0.1	1.4 ± 0.3	27 ± 2
	2	0.04 ± 0.09	1 ± 2	0 ± 1
	3	0.6 ± 0.2	1.9 ± 0.3	8 ± 2
	4	0.1 ± 0.1	1.6 ± 0.3	2 ± 1

*K*_*c*||_ is the cubic anisotropy constant and *K*_*u*||_ the uniaxial anisotropy constant. The interlayer exchange coupling constant *A*_ex_ has a common value for both layers.
