# Supplementary material for: Spin pumping in magnetic trilayer structures with an MgO barrier
Source: Sci Rep. 2016 Oct 18;6:35582. doi: 10.1038/srep35582 (PMC5067716; doi:10.1038/srep35582)
Supplement: Supplementary Information [file srep35582-s1.pdf]

Spin pumping in magnetic trilayer structures with an MgO barrier
Supplementary Information

A. A. Baker,[1,2] A. I. Figueroa,[2] D. Pingstone,[3] V. K. Lazarov,[3] G. van der Laan,[2] and T. Hesjedal[1,a]

[1] *Department of Physics, Clarendon Laboratory, University of Oxford, Oxford, OX1 3PU, United Kingdom*

[2] *Magnetic Spectroscopy Group, Diamond Light Source, Didcot, OX11 0DE, United Kingdom*

[3] *Department of Physics, University of York, Heslington, York, YO10 5DD, United Kingdom*

(Dated: 9 September 2016)

[a] Corresponding author. Email: thorsten.hesjedal@physics.ox.ac.uk

S1. KITTEL EQUATION

The condition for ferromagnetic resonance is derived using the macro-spin approximation, wherein all spins are assumed to undergo coherent precession. This approach implicitly neglects the contribution of the exchange interaction to the magnetisation dynamics. Further, it is not strictly true that all spins precess coherently in a material undergoing FMR, particularly in the case of coupled multilayers or patterned samples. However, this approximation is very useful as it allows the resonance condition to be derived using a simple variational approach that captures the key features of FMR.

In the case of small perturbations, and including a correction for Gilbert damping, the resonance frequency is found using[1]:

$$\left(\frac{\omega}{\gamma}\right)^2 = \frac{1+\alpha^2}{\left[M\sin(\theta)\right]^2}\left[\frac{\partial^2 F}{\partial\theta^2}\frac{\partial^2 F}{\partial\phi^2} - \left(\frac{\partial^2 F}{\partial\theta\partial\phi}\right)^2\right] , \tag{1}$$

with $\gamma = g\mu_\mathrm{B}/\hbar$ the gyromagnetic ratio. Again restricting the magnetisation to lie in-plane, this reduces to[1]:

$$\begin{aligned}\left(\frac{\omega}{\gamma}\right)^2 = \left(1+\alpha^2\right) & \Big[\mu_0 M + \mu_0 H\cos\left[\phi_\mathrm{M}-\phi_\mathrm{H}\right] + \frac{K_\mathrm{C}}{2M}\left(3+\cos\left[4\left(\phi_\mathrm{M}-\phi_\mathrm{C}\right)\right]\right) + \\ & \frac{K_\mathrm{U}}{M}\left(1-\cos\left[2\left(\phi_\mathrm{M}-\phi_\mathrm{U}\right)\right]\right) + \frac{A_\mathrm{ex}}{Mt}\cos\left[\phi_\mathrm{M1}-\phi_\mathrm{M2}\right]\Big] \\ & \Big[\mu_0 H\cos\left[\phi_\mathrm{M}-\phi_\mathrm{H}\right] + \frac{2K_\mathrm{C}}{M}\left(\cos\left[4\left(\phi_\mathrm{M}-\phi_\mathrm{C}\right)\right]\right) - \\ & \frac{2K_\mathrm{U}}{M}\cos\left[2\left(\phi_\mathrm{M}-\phi_\mathrm{U}\right)\right] + \frac{A_\mathrm{ex}}{Mt}\cos\left[\phi_\mathrm{M1}-\phi_\mathrm{M2}\right]\Big] .\end{aligned} \tag{2}$$

This is the Kittel equation[2] that is used to extract material parameters from the resonant fields measured in an FMR experiment, and A_ex is the exchange coupling.

S2. TRANSMISSION ELECTRON MICROSCOPY

Figure S1 shows a comparison of the cross-sectional view of two Ni/MgO(x)/CoFe/MgO(001) magnetic trilayer structures, where x is 1 nm (left-hand side) and 2 nm (right-hand side).

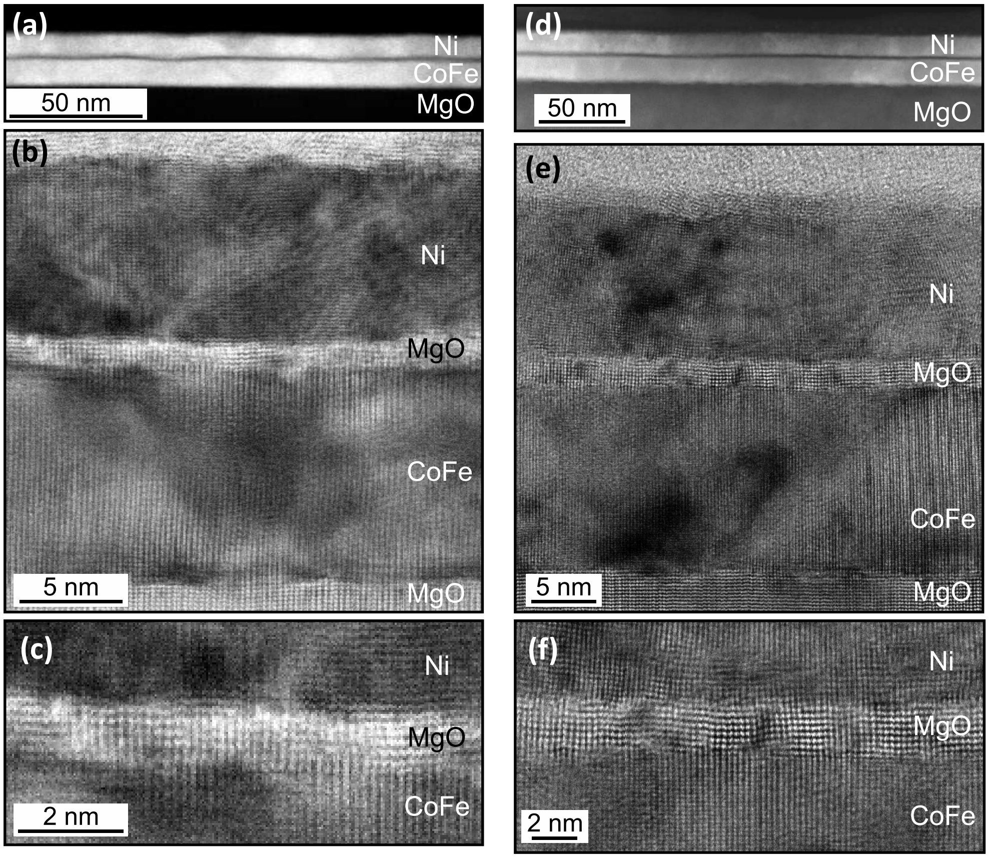

FIG. S1. **Cross-sectional TEM view of two Ni/MgO(x)/CoFe/MgO(001) magnetic trilayer structures with different MgO barrier thicknesses.** On the left-hand side (**a-c**) the results for a 1-nm-thick MgO barrier are shown. On the right-hand side (**d-f**), the MgO barrier thickness is 2 nm (cf. with Fig. 1 in the main text). (**a,d**) Low-magnification HAADF image of the magnetic trilayer structures showing uniform electrode thickness and varying MgO interlayer thickness. (**b,e**) High-resolution bright-field (BF) STEM images showing the atomic structure of the substrate, electrodes and barrier in [010] projection. (**c,f**) Atomic resolution BF-STEM of the Ni/MgO/CoFe interface region, showing structured 1 nm and 2-nm-thick MgO barriers along the (001) direction, respectively.

S3. FERROMAGNETIC RESONANCE MEASUREMENTS

VNA-FMR measurements were performed to determine values for the interlayer exchange coupling and magnetocrystalline anisotropy parameters for all samples. Figure S2 shows an example field-frequency transmission map, measured for the sample with $t_{\mathrm{MgO}} = 1$ nm, with the field along the magnetic (a) easy and (b) hard axis. Despite the static coupling causing the two layers to switch together in measurements of hysteresis loops, there are still two distinct resonance modes, indicating that the magnetodynamics of the heterostructure are not as strongly bound. Fits to resonance fields were performed as a function of excitation frequency and magnetization alignment using the Kittel equation, including the field due to static exchange coupling.

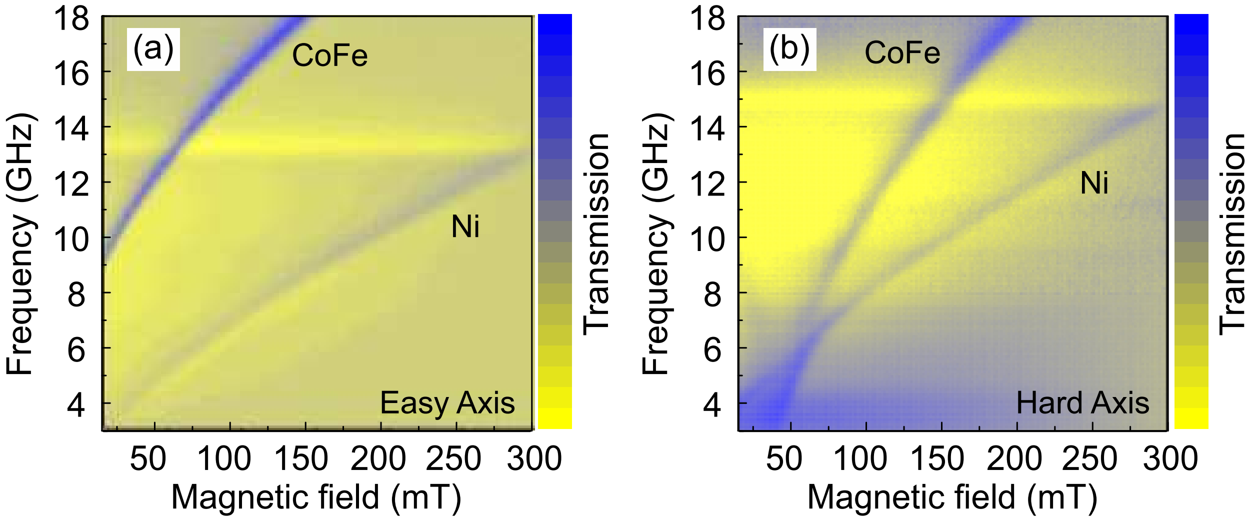

FIG. S2. **Lab-based FMR measurements.** VNA-FMR field-frequency transmission map for the sample with $t_{\mathrm{MgO}} = 1$ nm, and the magnetic field applied along (a) the easy and (b) the hard axis.

REFERENCES

[1]Farle, M. Ferromagnetic resonance of ultrathin metallic layers. *Rep. Prog. Phys.* **61**, 755 (1998).

[2]Kittel, C. On the theory of ferromagnetic resonance absorption. *Phys. Rev.* **73**, 155 (1948).
